# Cardiac systole is associated with enhanced go responding in an orthogonalized go/nogo task

**DOI:** 10.1038/s41598-026-52930-9

**Published:** 2026-05-21

**Authors:** Filippo Queirazza, Joana Carvalheiro, Marios G. Philiastides

**Affiliations:** 1 School of Health and Wellbeing, University of Glasgow, Glasgow, G12 0XH UK; 2 School of Psychology and Neuroscience, University of Glasgow, Glasgow, G12 8QB UK

**Keywords:** Interoception, Cardiac cycle, Decision-making, fMRI, Go/nogo task, Motivational biases, Neuroscience, Psychology, Psychology

## Abstract

Action selection is traditionally thought to be governed by dual top-down control systems: a fast, affect-dependent controller and a slow, experience-driven controller. Emerging evidence indicates that bottom-up cardiac phase signals also contribute to action selection by facilitating (i.e., go) or suppressing (i.e., nogo) motor responses. Despite growing interest in cardiac-brain interactions, no prior study has examined whether cardiac phase effects on feedback-driven action decisions are modulated by appetitive and aversive motivational states. We conducted a secondary analysis of a go/nogo functional magnetic resonance imaging (fMRI) dataset (*n* = 29), retrospectively determining the onset of decision cues relative to physiologically defined cardiac phases. We tested competing behavioural and computational hypotheses proposing that cardiac phase modulated action selection via motivational action biases, or through a general, affect-independent prepotent urge to act. Additionally, we performed an exploratory fMRI analysis to investigate neural activity underlying action decisions as a function of cardiac phase. Converging behavioural and computational evidence showed that systole was associated with a higher likelihood of go responding, independent of motivational action biases. Preliminary fMRI results further suggested that systolic facilitation of action was accompanied by diminished neural activity supporting action inhibition. Together, these findings extend existing evidence that cardiac phase impacts action decisions.

**Supplementary Information:**

The online version contains supplementary material available at 10.1038/s41598-026-52930-9.

## Introduction

Action selection is a fundamental component of adaptive decision-making and refers to the ability to select optimal (or approximately optimal) motor responses to environmental demands^1^. In experimental settings, action decisions are typically operationalised as the cue-triggered, goal-directed execution (i.e., go) or withholding (i.e., nogo) of a motor response^2,3^.

Influential theories on the computational underpinnings of action decisions have converged on the proposal of a dual system of complementary and partially overlapping action controllers^4–8^. While one control system (also known as instrumental system) is experience-driven and flexibly learns stimulus-response mappings based on contingent outcomes^9,10^, the other controller (also known as Pavlovian system) is affect-dependent and prescribes conditioned responses based on the motivational value of expected outcomes^11^. Accordingly, these motivational action biases rigidly instruct go responses to the prospect of a reward (i.e., appetitive action bias) and nogo responses to the prospect of a punishment (i.e., aversive action bias).

Complementing the dual-process model of action decisions, recent work in the field of interoception furnished initial evidence that bottom-up physiological signals also play a central role in guiding voluntary and involuntary responses both in the oculomotor^12,13^ and somatomotor^14–28^ domain. Interoception is the sensing of the internal physiological state of the body^29^. Interoception research has gathered increasing evidence that internal bodily states shape cognitive, perceptual, and emotional appraisals^30–39^. Notably, the anatomical and functional organisation of the neural and humoral pathways relaying interoceptive information to the brain has been well characterised^31^.

Cardiovascular afferents have been particularly leveraged to investigate the wide-ranging influence of interoceptive signals on mental processes and behaviour. Information about the timing and strength of cardiac contractions is periodically transmitted to the brain via multiple concurrent pathways^40^. While a primary route involves the activation of the baroreceptors located in the aortic arch and carotid sinuses, which respond to arterial stretch during ventricular systole to regulate blood pressure through a brainstem reflex (i.e., baroreflex), this represents only one component of the heart-brain axis^41,42^. Emerging evidence suggests that cardiac-brain interactions are supported by a broader range of afferent signals, including direct mechanosensory transduction and somatosensory inputs, which may operate independently of classical baroreceptor timing models^40^.

Consequently, the systolic phase, defined by ventricular contraction and the resulting arterial pressure increase, represents a period of complex, multimodal signalling that informs the brain of the heart’s state, whereas the diastolic phase corresponds to the period of ventricular relaxation and relative afferent quiescence. Importantly, in addition to their role in homeostatic control, cardiovascular signals have been shown to modulate emotion perception and appraisal^36–39^, auditory perception^43^, nociception^44^, somatosensory perception^45–47^, sense of agency^48^ and memory recall^30^.

Crucially, phasic fluctuations in cardiovascular arousal have been demonstrated to influence action decisions. In the motor domain, conflicting evidence has been reported that action execution can be either facilitated^12–20^ or inhibited^21–23^ during systole. Methodological differences across studies, including experimental design, task demands, and the operationalisation of cardiac phases, may account for these discrepant empirical findings.

More recently, research has begun to examine cardiac phase effects on action decisions in the context of feedback-driven learning, rather than on action selection per se. Preliminary evidence suggests that cardiac phase modulates the evaluative processing of action feedback. Using EEG during a gambling task, Kimura et al. demonstrated that outcome evaluation was enhanced when reward feedback occurred during systole, as reflected by larger feedback-related EEG amplitudes^49^. Importantly, this effect was selective for gains rather than losses, suggesting a valence-specific amplification of reward processing during systole. Extending this work to instrumental learning, the same author further showed that cardiac phase influenced asymmetries in value updating, such that learning rates for positive prediction errors exceeded those for negative prediction errors during systole, with no such asymmetry observed during diastole^50^.

Despite accumulating evidence for cardiac-related modulation of emotional appraisal^36–39^ and motivationally salient feedback^49,50^, no prior study has provided a comprehensive mechanistic account linking cardiac phase signals to action decisions as a function of appetitive and aversive motivational states. Moreover, most existing work on cardiac phase and motor behaviour has focused on learning-independent action control, that is, on the online selection, initiation, or inhibition of actions based on current task demands and internal states, rather than on changes in choice behaviour driven by feedback-dependent learning. It therefore remains unclear whether, in the context of learning-dependent action control, cardiac phase signals bias action decisions indirectly, via feedback-dependent motivational action biases (i.e., reward-driven action invigoration or punishment-driven action inhibition), or more directly, via affect-independent modulation of action invigoration or inhibition.

To address these open questions in interoception research, we conducted a secondary analysis of an orthogonalized go/nogo fMRI dataset originally collected to probe the neurocomputational underpinnings of the dual process model of decision-making^51^. We retrospectively retrieved the physiological cardiac timing of the task’s decision cues and subsequently fitted regression and computational models explicitly testing competing hypotheses that cardiac phase was linked to action selection indirectly, via feedback-dependent appetitive and aversive motivational action biases or directly, through learning-independent, affect-neutral modulation of motor responding. We also conducted exploratory analyses of the underlying neural correlates supporting action invigoration and inhibition as a function of cardiac phase.

Our findings extend current understanding of the neurocomputational pathways linking physiologically defined cardiac afferent signals to action decisions in feedback-driven learning and pave the way to future interoceptive research work investigating the cardiac-related neurocomputational mechanisms of decision-making.

## Methods

### Sample

This study is a secondary analysis of a previously published neuroimaging dataset^51^. Out of the 45 participants recruited for the original fMRI experiment, cardiac pulse recordings were available for 29 participants (25 females) due to technical constraints, specifically the unavailability of an MRI-compatible pulse sensor. Physiological data were used to retrospectively retrieve timings of the cardiac cycle. The mean age of the sample was 22.2 years (SD = ± 2.87) and the age range was between 18 and 32 years. A power analysis was not performed as this study was an exploratory analysis of secondary data. Nevertheless, the sample size is comparable to that of previous studies investigating cardiac phase effects on action control^18,52^ and choice behaviour^49,53^. Participants were recruited from the School of Psychology and Neuroscience research subjects’ pool. All participants gave written, informed consent. The original study protocol was approved by the College of Science and Engineering ethics committee (300160098) at the University of Glasgow, and all experiments were performed in accordance with relevant guidelines and regulations.

### Behavioural task

**Fig. 1:**
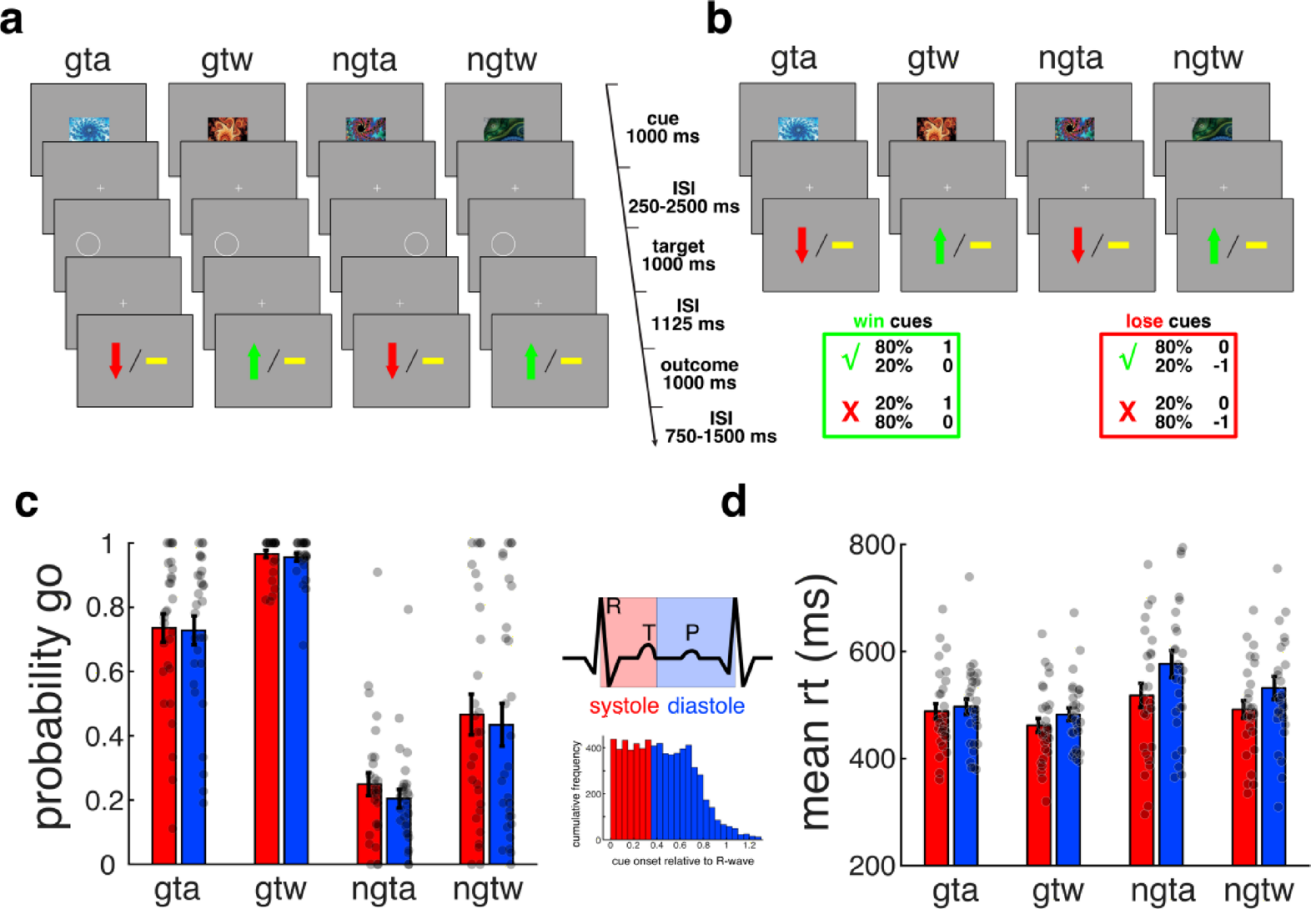
Task design and behaviour. **a-b** Mixed (**a**) and Pavlovian only (**b**) trials. Probabilistic reinforcement schedules for win and lose cues are shown in the green and red box respectively. **c-d** Cue-wise proportions of go responses (**c**) and average response times (**d**) as a function of cardiac phase. Error bars denote standard error of the mean. Translucent black dots represent individual subjects. Inset shows schematic of the cardiac cycle where the systole indicates ventricular contraction and the diastole denotes ventricular relaxation. Histogram plot shows population-level cumulative frequency of trials as a function of cardiac phase. gta: go to avoid losing; gtw: go to win; ngta: nogo to avoid losing; ngtw: nogo to win.

The behavioural task employed during fMRI scanning was a modified version of the validated orthogonalized go/nogo task^54^, which is a widely used behavioural paradigm to probe the effects of motivational biases and instrumental control on action selection. The task comprised four blocks, each containing 40 mixed trials (i.e., instrumental and Pavlovian) and 20 Pavlovian-only trials (60 trials per block and 240 trials in total; Fig. 1a-b). In each block, mixed trials were randomly interspersed with Pavlovian-only trials. In mixed trials, participants were initially presented with a fractal cue, followed by a target detection phase and probabilistic outcome feedback (Fig. 1a). Each of the four fractal cues indicated a unique association of action requirement (i.e., go vs. nogo) and outcome valence (i.e., win vs. lose), resulting in four conditions (i.e., go to win / nogo to win / go to avoid losing / nogo to avoid losing), which were randomised across participants. During the target detection phase participants were shown a circle on either the right- or left-hand side of the screen and target positions were counterbalanced across trials within each block. Right or left button presses had to match the cue location on the screen. Outcomes included an upward pointing green arrow (1 point) or a horizontal yellow bar (0 point) in the win trials and a downward pointing red arrow (−1 point) or a horizontal yellow bar (0 point) in the lose trials. Response-outcome contingencies were probabilistic (Fig. 1b). In Pavlovian only trials, no motor response was required, and the presentation of a fractal cue was followed by outcome feedback (Fig. 1b), which was selected as if the participant had made the correct response.

Participants were instructed to maximise correct responses (i.e., go or nogo) by trial and error and were informed of the probabilistic nature of the task but not of the response-outcome contingencies. They could win up to £10 depending on their task performance. All participants practised an example block of the task before fMRI scanning to familiarise themselves with the speed requirements of the task. The task was programmed using Presentation^®^ (Neurobehavioural Systems) stimulus delivery software.

### fMRI data acquisition

Neuroimaging data were acquired using a 3-Tesla Siemens TIM Trio MRI scanner (Siemens, Erlangen, Germany) with a 12-channel head coil. Cushions were placed around the head of participants to minimize head motion. Neuroimaging data included a high-resolution T1-weighted structural image (1 mm isotropic voxels, 128 axial slices, TI = 900 ms, TR = 2300 ms, TE = 2.96 ms, flip angle=90°), a T2*-weighted echo planar imaging (EPI) functional scan (2 mm isotropic voxels, 68 axial slices, TR = 2000 ms, TE = 26 ms, flip angle = 60°, multiband acquisition factor 2), phase and magnitude fieldmaps (3.3 × 3.3 × 3 mm voxels, 46 axial slices, TR = 488 ms, short TE = 4.92 ms, long TE = 7.38 ms) for EPI distortion correction. Slice orientation was tilted − 30° from the AC-PC plane to reduce susceptibility induced signal drop-out^55^.

### Cardiac data acquisition and analysis

Cardiac pulse data were collected during fMRI scanning using a dedicated MRI-compatible pulse sensor (Siemens Trio). The PhysIO toolbox was used to preprocess peripheral physiological recordings and retrieve onset timings of the R-wave peaks^56^. Briefly, the PhysIO toolbox implements a peak detection algorithm to recover the time events of the R-wave peaks from the raw cardiac time series. Temporal alignment between the cardiac recordings, fMRI time series, and behavioural task events was achieved using a scanner-generated trigger pulse that was recorded concurrently in both the physiological and task data streams. This trigger provided a common temporal reference for subsequent synchronization.

Trials were sorted based on whether the onset of cue presentation occurred during the systolic or diastolic phase. Systole was defined as the time between the onset of the R-wave peak and the end of the T wave (i.e., 350 ms after the R-wave peak) (inset Fig. 1). Diastole was defined as the time between the offset of the systole and the onset of the next R-wave peak. While according to the classical arterial baroreceptor framework cortical effects are frequently reported in the 150–250 ms post-R-peak window in humans, defining a single “functionally relevant” interval is not straightforward. Our decision to employ a broader, physiologically grounded systolic window was intended to reflect the transient haemodynamic and afferent state initiated during ventricular contraction, which may engage multiple levels of cardiac-brain signalling, rather than to rigidly isolate a specific cortical latency window^40^.

It is also worth noting that given the retrospective nature of the dataset and inter- and intra-individual variability in ECG morphology and cardiac timing, imposing a fixed post-R-peak latency window would require strong assumptions regarding uniform cortical timing across participants and trials and could introduce systematic misalignment.

Of note, the unequal duration of systole and diastole resulted in a greater number of trials being classified as diastole.

### Behavioural analysis

The main goal of the behavioural analyses was to investigate the relationship between the physiologically defined cardiac cycle and action decisions. Specifically, the behavioural analyses aimed to ascertain whether cardiac phase was linked to action decisions via motivational biases on action selection.

Using the lme4 package in R (http://www.r-project.org), maximal by-subject generalised and loglinear mixed-effects models were conducted to test main and interaction effects of cardiac phase (i.e., systole vs. diastole) and outcome valence (i.e., win vs. lose cues) on action selection (i.e., go vs. no go)^57^. Statistical significance of the fixed effects was tested using the likelihood ratio test^57^. In cases of non-convergence, the random effects structure of the model was simplified by dropping correlation terms and random slopes.

To test the effects of the cardiac phase and outcome valence on action selection (i.e., go vs. nogo) and response times (RTs), we run the following mixed-effects regression models:

$${\text{logit(Response) = 1 + CardiacPhase * Valence + (1 + Valence | Subject)}}{\mathrm{.}}$$

$${\text{log(RT) = 1 + CardiacPhase * Valence + (1 + CardiacPhase * Valence | Subject)}}{\mathrm{.}}$$

Importantly, mixed-effects regression models estimate effects at the trial level using likelihood-based inference and explicitly account for within-subject dependence through random effects. As a result, they are well suited to unbalanced designs and are robust to unequal numbers of observations across conditions, such as the imbalance between systole and diastole trials. Although unequal trial counts may have reduced the precision of parameter estimates, they are unlikely to have biased the fixed-effect estimates of the regressors.

However, to further ensure robustness against unequal trial counts, we conducted a subsampling control analysis in which diastole trials were randomly subsampled to match systole trial counts within each participant. Mixed-effects models were refitted across 1000 subsampled datasets and the resulting distribution and confidence intervals of subsampled fixed-effect estimates were examined. It is important to note that the primary goal of the subsampling analyses was not to perform an inferential test, but to show that the direction and magnitude of the fixed effects remained stable when trial counts were equalised.

### Computational modelling

Computational models were fitted to the behavioural data to complement the behavioural analyses and test competing mechanistic accounts of the relationship between the physiologically defined cardiac cycle and action selection. In line with the behavioural analyses, we considered two hypotheses: that cardiac phase is linked to action decisions (i) either via a Pavlovian bias parameter on action weights (ii) or through an affect-independent, prepotent urge to select go responses.

#### Null model

The null model was the best fitting model from previous modelling work^51^. According to this model, action weight $\:\mathrm{W}$ determined the propensity$\:\:\mathrm{p}$ for action$\:\:\mathrm{a}\:\in\:(\mathrm{g}\mathrm{o},\mathrm{n}\mathrm{o}\mathrm{g}\mathrm{o})$ as follows:

$$\:p({a}_{go}^{t}\left|\:{cue}_{i}^{t}\right)=\:\sigma\left(\left({W}_{go}^{t}\left|{cue}_{i}^{t})-\:{(W}_{no-go}^{t}\right|{cue}_{i}^{t}\right)\right)\left(1-noise\right)+\:\frac{noise}{2}$$

where $\:{\upsigma\:}\left(\right)$ is a standard sigmoid function, superscript $\:\mathrm{t}$ stands for trial, subscript $\:\mathrm{i}$ indicates a specific cue and $\:\mathrm{n}\mathrm{o}\mathrm{i}\mathrm{s}\mathrm{e}$ is a free parameter that can vary between 0 and 1. The action weight $\:\mathrm{W}$ was updated according to the following equation:

$$\:W_{i}^{t} \left( {a^{t} |cue_{i}^{t} } \right)\left\{ {\begin{array}{*{20}l} {Q_{i}^{t} (a^{t} |\:cue_{i}^{t} ) + b + \:\pi \:V_{i}^{t} \left( {cue_{i}^{t} } \right)} & {if\:a = go} \\ {Q_{i}^{t} (a^{t} |\:cue_{i}^{t} )} & {else} \\ \end{array} } \right.$$

where the parameter $\:\mathrm{b}$ captures the go bias (i.e., the observed affect-independent, prepotent tendency to favour go over nogo responses), $\:\mathrm{Q}$ and $\:\mathrm{V}$ represent the instrumental and Pavlovian (expected) value respectively, the parameter $\:{\uppi\:}$ indexes the magnitude of the Pavlovian bias (i.e., the extent to which the Pavlovian valuation system influences instrumental responding). Given that the sign of the Pavlovian value $\:\mathrm{V}$ is determined by cue valence (i.e., positive for win cues and negative for lose cues), $\:\mathrm{V}$ increased the value of the go action weight during the win trials but decreased it during the lose trials.

The update equation for the Q value was a standard Rescorla-Wagner learning rule parameterised as follows:

$$\:Q_{i}^{t} (a^{t} |\:cue_{i}^{t} )\left\{ {\begin{array}{*{20}l} {Q_{i}^{{t - 1}} (a^{{t - 1}} |\:cue_{i}^{{t - 1}} ) + \:\alpha \:\:(\rho \:_{{rew}} r^{t} - \:Q_{i}^{{t - 1}} \left( {a^{{t - 1}} |\:cue_{i}^{{t - 1}} )} \right)} & {if\:cue = \: gtw \lor ngtw} \\ {Q_{i}^{{t - 1}} (a^{{t - 1}} |\:cue_{i}^{{t - 1}} ) + \:\alpha \:\:(\rho \:_{{pun}} r^{t} - \:Q_{i}^{{t - 1}} \left( {a^{{t - 1}} |\:cue_{i}^{{t - 1}} )} \right)} & {if\:cue = \: gta \lor ngta} \\ \end{array} } \right.$$

where $\:{\mathrm{r}}^{\mathrm{t}}\in\:(\mathrm{1,0},-1)$ represents the outcome, $\:{\upalpha\:}$ is the learning rate and $\:{\uprho\:}$ is an outcome sensitivity parameter allowing for differential scaling of reward ($\:{{\uprho\:}}_{rew}$) and punishment ($\:{{\uprho\:}}_{pun}$) outcomes.

The update equation for the Pavlovian value $\:\mathrm{V}$ was also a standard Rescorla-Wagner equation:

$$\:V_{i}^{t} \left( {cue_{i}^{t} } \right)\left\{ {\begin{array}{*{20}l} {V_{i}^{{t - 1}} \left( {cue_{i}^{{t - 1}} } \right) + \:\alpha \:\:\left( {\rho _{{rew}} r^{t} - \:V_{i}^{{t - 1}} \left( {cue_{i}^{{t - 1}} } \right)} \right)if\:cue = \: gtw \lor ngtw} \\ {\:V_{i}^{{t - 1}} \left( {cue_{i}^{{t - 1}} } \right) + \:\alpha \:\:\left( {\rho _{{pun}} r^{t} - \:V_{i}^{{t - 1}} \left( {cue_{i}^{{t - 1}} } \right)} \right)if\:cue = \: gta \lor ngta} \\ \end{array} } \right.$$

While the initial instrumental value $\:{\mathrm{Q}}^{0}$ of all cues was fitted as a free parameter, the initial Pavlovian value $\:{\mathrm{V}}^{0}$ of all cues was set to 0.

#### Cardiac phase-dependent models

To test whether cardiac phase was linked to action selection via Pavlovian biases, we specified a *cardioPav* model in which the Pavlovian bias parameter $\:\pi\:$ varied as a function of cardiac phase:

$$W_{i}^{t} \left( {a^{t} |cue_{i}^{t} } \right))\left\{ {\begin{array}{*{20}l} {Q_{i}^{t} (a^{t} |\:cue_{i}^{t} ) + b + \pi _{{sys}} V_{i}^{t} \left( {cue_{i}^{t} } \right)} & {if\:a = go \wedge cardiac = sys} \\ {Q_{i}^{t} (a^{t} |\:cue_{i}^{t} ) + b + \pi _{{dia}} V_{i}^{t} \left( {cue_{i}^{t} } \right)} & {if\:a = go \wedge cardiac = dia} \\ {Q_{i}^{t} (a^{t} |\:cue_{i}^{t} )} & {else} \\ \end{array} } \right.$$

Alternatively, to test whether cardiac phase was directly linked to a general tendency to act, we specified a *cardioGo* model in which the go bias parameter $\:b$ varied as a function of cardiac phase:

$$\:W_{i}^{t} (a^{t} |\:cue_{i}^{t} )\left\{ {\begin{array}{*{20}l} {Q_{i}^{t} (a^{t} |\:cue_{i}^{t} ) + b_{{sys}} + \pi \:V_{i}^{t} \left( {cue_{i}^{t} } \right)} & {if\:a = go \wedge \:cardiac = sys} \\ {Q_{i}^{t} (a^{t} |\:cue_{i}^{t} ) + b_{{dia}} + \pi \:V_{i}^{t} \left( {cue_{i}^{t} } \right)} & {if\:a = go \wedge \:cardiac = dia} \\ {Q_{i}^{t} (a^{t} |\:cue_{i}^{t} )} & {else} \\ \end{array} } \right.$$

These two competing models were compared against the null model to assess whether incorporating cardiac phase information improved model fit. Moreover, within-subjects comparison of the cardiac phase-specific parameters was conducted using a two-sample paired t-test.

To preserve the parameters’ natural bounds, log $\:({\uprho\:},\:{\uppi\:})$ and logit $\:(\mathrm{n}\mathrm{o}\mathrm{i}\mathrm{s}\mathrm{e},\:{\upalpha\:})$ transforms of the parameters were implemented in all models. We set the initial value of the free parameters’ $\:(\mathrm{n}\mathrm{o}\mathrm{i}\mathrm{s}\mathrm{e},\:{\upalpha\:},\:\mathrm{b},{\mathrm{b}}_{sys},{\mathrm{b}}_{dia},\:{\uprho\:},\:{{\uprho\:}}_{rew},\:{{\uprho\:}}_{pun},\:{\uppi\:},{{\uppi\:}}_{sys},{{\uppi\:}}_{dia},\:{\mathrm{Q}}^{0})$ prior means in their native space to (0.5, 0.5, 0, 0, 0, 1, 1, 1, 1, 1, 1, 0) and their prior variances to 100.

### Model fitting and validation

A hierarchical type II maximum likelihood fitting procedure was implemented to fit the computational models to the behavioural data^51,58^. In this framework, group-level prior parameters are estimated from the data rather than fixed a priori, allowing individual parameter estimates to be regularised in a principled manner while accommodating between-subject variability. Maximum a posteriori estimates of the parameters were computed to prevent overfitting and avoid noisy parameters estimates since poorly constrained parameters are shrunk towards the group-level priors.

Prior parameters $\:{\upeta\:}$ were optimised by performing k iterations of an expectation-maximization routine until convergence. Briefly, at each iteration k, in the expectation step we optimised the log likelihood of observed data Y with respect to the distribution over the parameters $\:{\uptheta\:}$ holding prior parameters $\:{\upeta\:}$ fixed:

$$\:{q}^{k}\left(\theta\:\right)=p\left(\theta\:\right|Y,{\eta\:}^{k-1})$$

We used a Laplace approximation for $\:{q}^{k}\left(\theta\:\right)\:\sim\:N=({m}^{k},{s}^{k})$ and for each subject i updated the mean m and variance s of the normal distribution as follows:

$$m_{i}^{k} = \mathop {\arg \max }\limits_{\theta } p(Y_{i} ,\theta _{i} |\eta ^{{k - 1}} )$$

$$\:{s}_{i}^{k}=\:{\left(\frac{{\partial\:}^{2}p({Y}_{i},{\theta\:}_{i}|{\eta\:}^{k-1})}{\partial\:{\theta\:}_{i}^{2}}\left.\vphantom{\frac{\partial^{2}p(Y_i,\theta_i|\eta^{k-1})}{\partial\theta_i^{2}}}\right|_{\theta_i=m_i^{k}}\right)}^{-1}$$

Subsequently in the maximization step we optimised the log likelihood with respect to prior parameters $\:\eta\:$ holding the distribution over the parameters $\:{\uptheta\:}$ fixed:

$$\:{\eta\:}_{m}^{k}=\frac{1}{N}\stackrel{N}{\sum\:_{i}}{m}_{i}^{k}$$

$$\:{\eta\:}_{s}^{k}=\frac{1}{N}\stackrel{N}{\sum\:_{i}}({m}_{i}^{k}{)}^{2}+{s}_{i}^{k}-{\eta\:}_{m}^{k}$$

The 20% bend correlation coefficient between observed and model-predicted group-level trial-wise probabilities of go action was computed to verify the model’s goodness of fit. Parameter recovery was performed to test whether the parameters of the best fitting model were identifiable.

Importantly, the hierarchical type II maximum likelihood fitting procedure naturally accommodated unequal numbers of systole and diastole trials by estimating parameters at the trial level and regularising subject-specific estimates via group-level priors. Participants with fewer systole trials contributed greater uncertainty to systole-specific parameters, which were correspondingly shrunk towards the group mean. This ensured that cardiac phase effects were estimated conservatively. As a result, although unequal trial counts may have reduced the precision of the cardiac phase parameter estimates, they are unlikely to have affected the validity or direction of the observed effects.

### Model comparison and simulation

Random-effects Bayesian model comparison was performed to assess the predictive performance of the candidate models and select the best fitting model^59^. Briefly, we estimated and compared the models’ protected exceedance probabilities, which quantify the probability that any one model is more frequent than the others, above and beyond chance^59^.

To evaluate generative performance of the best fitting model^60^, we correlated observed and simulated trial- and cue-wise group-level probabilities of the go action using 20% bend correlation. Synthetic data were generated by simulating the task 100 times. For each simulation we resampled individual fitted parameters without replacement and then simulated subject-wise go and nogo responses^51^.

### fMRI data pre-processing and analysis

The fMRI pre-processing steps were previously described in ^51^. To identify neural correlates of go and nogo responses, whole brain statistical analyses of the fMRI data were performed using a multilevel mixed-effects approach as implemented in FLAME1 (FSL)^61^. In the first-level design matrix, the regressors of interest were four unmodulated boxcar functions, each aligned with cue onset (i.e., go to win / nogo to win / go to avoid losing / nogo to avoid losing). Additional regressors included two boxcar regressors for RT-modulated go and unmodulated nogo responses in the target detection phase, one modulated boxcar regressor modelling outcome (i.e. +1 for rewards, 0 for neutral outcomes and − 1 for punishments) and one unmodulated regressor for late response trials. Regressors were convolved with a double gamma hemodynamic response function. Moreover, six cardiac and six motion (three translations and three rotations) regressors were included in the design matrix as regressors of no interest. The six cardiac confound regressors were obtained by means of a 3rd order Fourier expansion of the pre-processed cardiac time series^62^.

At the first level, linear contrasts of the cue-wise regressors were carried out to test the main effects of action (go > nogo cues) and inaction (nogo > go cues). As a sanity check, a single contrast combining all cardiac regressors was set up to retrieve brain activations related to the cardiac cycle.

At the second and third level a one-sample t-test was conducted on the lower-level contrasts of the parameter estimates to account for the between-blocks (second level) and between-subjects (third level) random effects. The resulting Z statistic images were thresholded using a cluster-defining threshold of Z > 3.1 and an FWE-corrected significance threshold of *p* = 0.05.

### Time course analysis of fMRI data

The raw pre-processed BOLD time courses were extracted from the significant clusters identified in the action and inaction contrasts and BOLD percent signal change within each of these clusters was estimated according to the following formula^51,63^:

$$BOLD\% \:signal\:change^{t} = \left( {\frac{{BOLD^{t} - \:BOLD\:baseline}}{{\overline{BOLD} }}} \right)$$

where superscript t indicates time point, BOLD baseline is the average BOLD signal over the 4 s preceding the onset of the decision phase and $\:\overline{BOLD}$ is the mean BOLD signal across all time points.

The cluster-wise BOLD traces were averaged by cardiac phase and within-subject comparison (i.e., systole vs. diastole) of peak BOLD activity was performed using a two-sample paired t-test. Multiple comparison correction was performed using the maximum-statistic permutation method (*n* = 1000), which controls the family-wise error rate by comparing observed test statistics with a permutation-derived null distribution of the largest statistic expected across clusters under the null hypothesis^64^.

Because BOLD responses were averaged within condition prior to statistical testing, the unequal number of systole and diastole trials was potentially more problematic for the fMRI analyses. To address this concern, we conducted additional control analyses in which diastole trials were randomly subsampled within each participant to match the number of systole trials. This procedure was repeated iteratively (*n* = 1000), and for each permutation we estimated the within-subject systole - diastole contrast averaged across participants. The resulting distribution and confidence intervals of subsampled contrast estimates were examined to assess whether the direction and magnitude of the contrast estimates remained stable when trial counts were equalised.

## Results

### Cue presentation at systole was associated with greater likelihood of go responding

On average, the probability of observing a go response was higher when cues were presented during systole (go to win: 96.6% ± 6%; nogo to avoid losing: 24.9% ± 29%; go to avoid losing: 73.6% ± 24%; nogo to win: 46.6% ± 34%) compared to diastole (go to win: 95.6% ± 6%; nogo to avoid losing: 20.3% ± 15%; go to avoid losing: 72.8% ± 24%; nogo to win: 35.9% ± 36%) (Fig. 1c). Consistent with this pattern, mean response times were faster following cue presentation at systole (go to win: 462 ± 70 ms; nogo to avoid losing: 518 ± 121 ms; go to avoid losing: 488 ± 75 ms; nogo to win: 491 ± 87 ms) than at diastole (go to win: 482 ± 64 ms; nogo to avoid losing: 576 ± 134 ms; go to avoid losing: 497 ± 77 ms; nogo to win: 532 ± 116 ms) (Fig. 1d). Owing to the unequal duration of cardiac phases, a significantly greater proportion of trials were classified as diastole (i.e., 58% ± 7%) than systole (t_28_ = 6.24, *p* < 0.001) (inset Fig. 1).

The results of the mixed-effects regression models confirmed and extended the descriptive statistics. There was a statistically significant main effect of cardiac phase (β = 0.13, *p* = 0.04) and outcome valence (β = -1.34, *p* < 0.001) on action selection but no significant interaction (β = − 0.02, *p* = 0.85). A similar pattern was observed for response times, with a marginal main effect of cardiac phase (β = 0.04, *p* = 0.055) and a significant main effect of outcome valence (β = − 0.05, *p* = 0.035), but no significant interaction (β = 0.02, *p* = 0.484).

Importantly, the significant fixed-effect estimates remained qualitatively unchanged in the sensitivity analyses that controlled for unequal trial counts. For action selection, the subsampled estimates were: cardiac phase ($\:\overline{\beta}$= 0.14, 95% CI = [0.09 0.19]), outcome valence ($\:\overline{\beta}$ = −1.33, 95% CI = [−1.4 −1.26]) and their interaction ( $\:\overline{\beta}$= − 0.05, 95% CI =[−0.15 0.05]). For response times, subsampled estimates were: cardiac phase ($\:\overline{\beta}$= 0.04, 95% CI = [0.03 0.05]), outcome valence ($\:\overline{\beta}$ = − 0.05, 95% CI = [−0.06 − 0.04]) and their interaction ($\:\overline{\beta}$= 0.02, 95% CI =[−0.004 0.04]). These findings indicate that the direction and magnitude of the fixed-effect estimates were robust to trial count imbalance and were not driven by unequal numbers of systole and diastole trials.

Taken together, these results show that participants were generally more likely to produce (marginally faster) go responses when decision cues were presented during the systolic phase of the cardiac cycle, independent of appetitive or aversive motivational biases. In line with previous findings^51^, motivational biases also exerted a significant main effect on action decisions: reward expectation invigorated and sped up instrumental responding, whereas punishment expectation suppressed and slowed down responding.

Given the observed association between cardiac phase and action selection, we capitalised on computational modelling to test competing hypotheses regarding the latent cognitive mechanisms through which cardiac phase is linked to action decisions.

### Cue presentation at systole was linked to stronger go bias

While the *null* model assumed no relationship between cardiac phase and action selection, the remaining two models posited that cardiac phase is linked to action selection indirectly, via appetitive and aversive motivational action biases (*cardioPav*), or directly, through affect-independent, prepotent go responding (*cardioGo*).

**Fig. 2:**
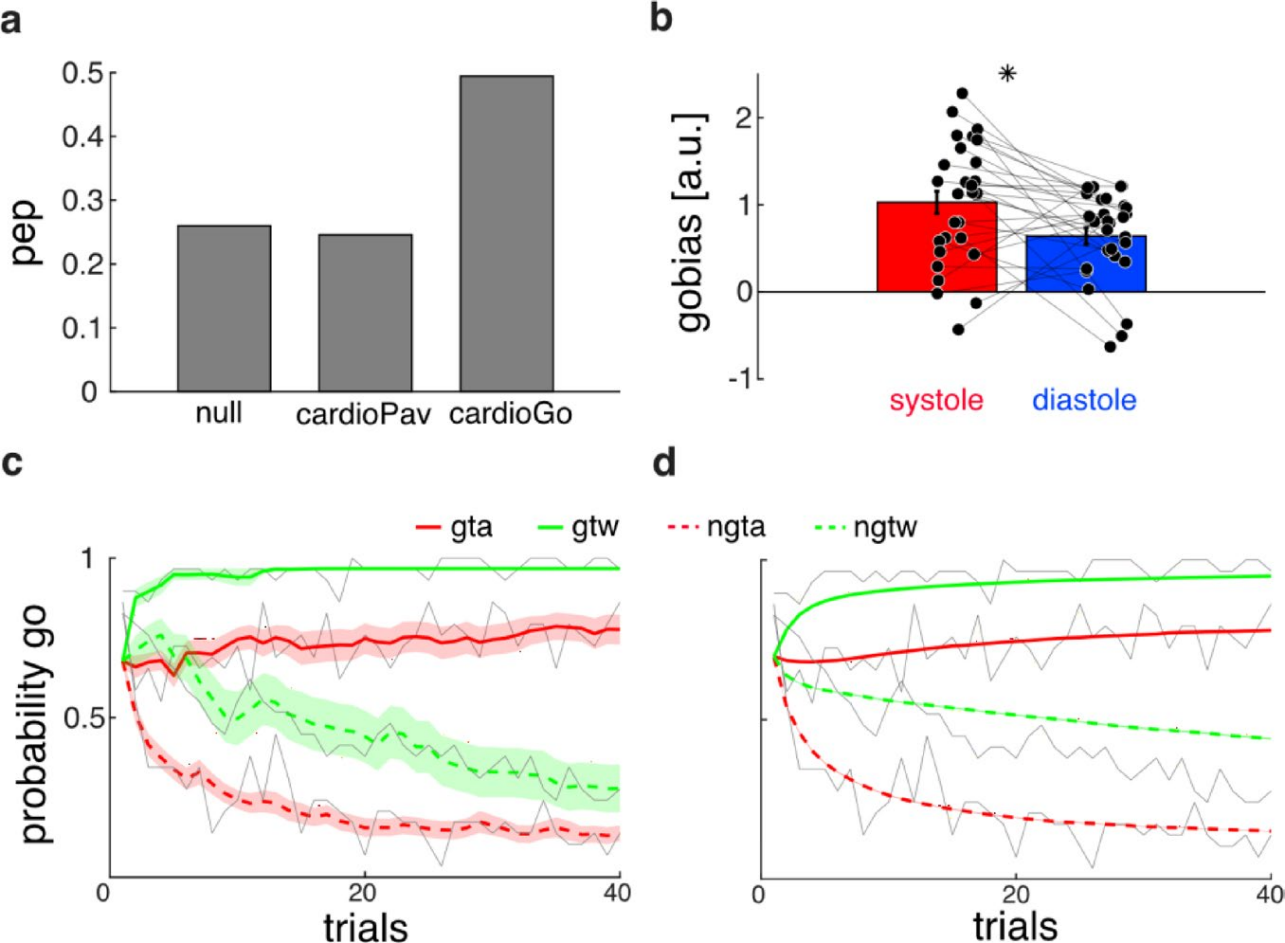
Computational modelling. **a** Bar plot showing protected exceedance probability (pep) (i.e., the probability that a model is more likely than any other model in the set, after accounting for the possibility that differences in model performance are due to chance) **b** Bar plot showing significantly greater within-subject estimates of the systolic go bias parameter. Error bars denote standard error of the mean (SEM) and black dots represent individual subjects. **c-d.** Fitted (c) and simulated (d) behavioural data from the best fitting model (i.e., cardioGo). Grey lines represent observed mean choice behaviour (i.e. trial-wise probability of choosing go action). Coloured shadings represent SEM. * *p* < 0.05.

Bayesian model comparison indicated that the *cardioGo* model was the best fitting model (Fig. 2a). Crucially, in the *cardioGo* model, the magnitude of the go bias parameter (and thus its influence on go responding) was significantly greater when decision cues were presented during systole compared with diastole (t_28_ = 2.57, *p* = 0.016) (Fig. 2b). Thus, the modelling results corroborated the behavioural finding that action invigoration at systole was not associated with motivational (Pavlovian) action biases but arose from a general, affect-independent urge to select the go response.

Importantly, the *cardioGo* model explicitly accounted for known asymmetries in gain-loss perception (e.g., loss aversion) by including two separate outcome sensitivity parameters for rewards and punishments, allowing differential scaling of positive and negative outcomes. Even after adjusting for these valence-dependent effects, the go bias parameter remained significantly higher during systole than diastole. This suggests that the observed cardiac phase effects on action selection were not driven by unmodelled gain-loss asymmetries.

To further validate the best fitting model, we assessed model fit and parameter recoverability. Fitted (r_158_ = 0.98, *p* < 0.001) and simulated (r_158_ = 0.97, *p* < 0.001) data of the best fitting model provided a good fit to observed choice behaviour (Fig. 2c-d). Fitted parameters were successfully recovered using the hierarchical type II maximum likelihood fitting routine (noise: r_27_ = 0.40, *p* = 0.03; α: r_27_ = 0.76, *p* < 0.001; b_sys_: r_27_ = 0.65, *p* < 0.001; b_dia_: r_27_ = 0.61, *p* = 0.001; ρ_rew_: r_27_ = 0.56, *p* = 0.002; ρ_pun_: r_27_ = 0.68, *p* < 0.001; π: r_27_ = 0.80, *p* < 0.001; Q^0^: r_27_ = 0.76, *p* < 0.001).

Overall, behavioural analyses and computational modelling converged on the common finding that systole was associated with action invigoration, independent of appetitive or aversive motivational biases.

### Cue presentation at systole was associated with larger right IFG (peak) BOLD activity supporting action inhibition

**Fig. 3:**
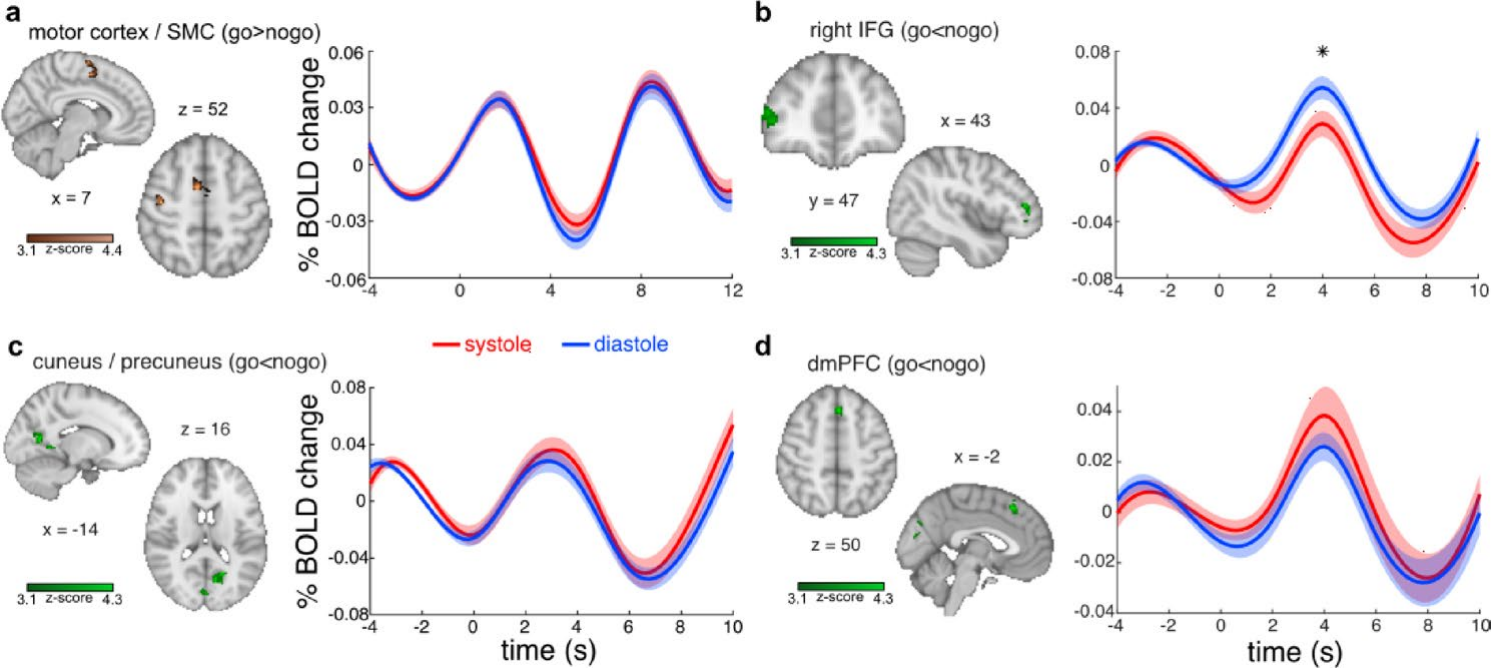
fMRI analysis. Cluster-wise BOLD traces locked to cue onset as a function of cardiac phase (systole: red; diastole: blue). Clusters denote significant activations linked to action (go > nogo; copper) and inaction (go < nogo; green) contrasts. Haemodynamic activity shows an effect of the cardiac cycle in the right IFG. MNI coordinates are shown. * uncorrected *p* < 0.05. SMC: supplementary motor cortex. IFG: inferior frontal gyrus. dmPFC: dorsomedial prefrontal cortex.

To explore the relationship between cardiac phase and neural correlates of action selection, we first identified brain activity underpinning go (i.e., action) and nogo (i.e., inaction) responses. While the main effect of action (i.e., go> nogo cues) was linked to significant activations in the predominantly right-sided supplementary motor cortex (peak Z score = 4.18; MNI space coordinates = 8,0,52; *p* < 0.05 FWE) (Fig. 3a) and right precentral gyrus (peak Z score = 4.38; MNI space coordinates = 40,-14,52; *p* < 0.05 FWE) (Fig. 3a), the main effect of inaction (i.e., nogo > go cues) revealed significant activations in the right inferior frontal gyrus (IFG) (peak Z score = 3.92; MNI space coordinates = 44,42,10; *p* < 0.05 FWE) (Fig. 3b), precuneal (peak Z score = 4.28; MNI space coordinates = 8, −40,0; *p* < 0.05 FWE) and cuneal cortex (peak Z score = 4.13; MNI space coordinates = 6, −84,36; *p* < 0.05 FWE) (Fig. 3c) and bilateral dorsomedial prefrontal cortex (dmPFC) (peak Z score = 4.16; MNI space coordinates = 0,26,48; *p* < 0.05 FWE) (Fig. 3d). As a sanity check, we ran a contrast representing the combined effect of the six cardiac regressors and retrieved neural activations reflecting underlying pulsatile cardiac activity in the cerebrospinal fluid, brain stem, in the vicinity of large blood vessels at the edge of the brain parenchyma and in interoceptive brain areas such as the anterior cingulate cortex and insula (Supplementary Fig. 1).

Next, we examined the relationship between cardiac phase and neural activity supporting action and inaction. Within-subject comparisons of cluster-wise raw BOLD activity revealed greater (peak) BOLD activity in the right IFG during diastole compared with systole (t_28_ = -2.07; p_uncorr_ = 0.048) (Fig. 3b). However, this effect did not survive multiple comparison correction (p_corr_ = 0.185). No significant cardiac phase-related differences were observed in the remaining clusters, including the motor/supplementary motor cortex (t_28_ = 0.313; p_uncorr_ = 0.75) (Fig. 3a), precuneal/cuneal cortex (t_28_ = 0.917; p_uncorr_ = 0.36) (Fig. 3c) and dmPFC (t_28_ = 0.904; p_uncorr_ = 0.37) (Fig. 3d).

Results from the subsampling sensitivity analyses were consistent with the full-data estimates across all clusters. Specifically, subsampled systole - diastole contrast estimates closely matched those obtained from the full dataset (right IFG: mean difference = − 0.0253, 95% CI [-0.0257, − 0.0250] vs. full-data estimate = − 0.0255; motor/supplementary motor cortex: 0.0030, 95% CI [0.0027, 0.0032] vs. 0.0030; precuneus/cuneus: 0.0111, 95% CI [0.0107, 0.0114] vs. 0.0109; dmPFC: 0.0123, 95% CI [0.0119, 0.0127] vs. 0.0123). These findings indicate that the observed fMRI effects were stable in direction and magnitude and were not driven by unequal numbers of systole and diastole trials.

Intriguingly, the neural findings seem to suggest that the systole-related action invigoration observed in the behavioural and computational data may in fact be associated with attenuated inhibitory action control at the neural level. Although these fMRI results are exploratory and should be interpreted with caution, as they did not survive correction for multiple comparisons, they provide the first empirical evidence linking cardiac phase to action selection via underlying, affect-independent action/inaction biases.

## Discussion

Since an early influential proposal that emotional experiences are grounded in bodily changes^65^, there has been growing recognition that peripheral physiological signals modulate perception, affect, cognition and action^33^. While information about the body’s changing visceral state, such as the dynamic changes in cardiovascular arousal, is a key determinant of the body’s homeostatic regulation, bottom-up visceral representations in the brain also subserve complex behaviours and higher-order mental processes^31,34^.

This study leveraged simultaneous cardiac pulse recordings acquired during an orthogonalized go/nogo fMRI paradigm to retrospectively retrieve decision cue onset times relative to the R-wave of the cardiac cycle, thereby enabling us to examine action decisions as a function of cardiovascular arousal. Each cue denoted the cross-product of outcome valence and action requirement, allowing us to disentangle the influence of motivational states on goal-directed action selection^11^.

Mixed-effects regression analyses of choice behaviour and response times revealed that decision cues presented during systole were associated with a significantly higher likelihood of (marginally faster) go responses. Notably, this effect was not moderated by the motivational valence of decision cues. Consistent with these behavioural findings, computational modelling indicated that the best fitting model captured a general, affect-independent prepotent tendency to go that was linked to the physiologically defined cardiac cycle. This model outperformed an alternative computational account in which the relationship between the cardiac cycle and go responding was mediated by cardiac phase-specific Pavlovian action biases. Crucially, within-subject estimates of the go bias parameter in the best fitting model were, on average, higher during systole than diastole, thus corroborating the behavioural evidence for enhanced action propensity at systole.

Consistent with our findings, Al et al. reported a facilitatory effect of systolic activity on cortical and corticospinal motor excitability^66^. Specifically, transcranial magnetic stimulation (TMS) applied over the motor cortex during systole elicited stronger motor-evoked potentials in the hand muscles, as well as enhanced TMS-evoked potentials in the motor cortex^66^. Moreover, during a motor pinch task, hand-muscle force was greater at systole than during diastole^66^. Complementary evidence comes from a study showing that, during a visual recognition memory task, participants exhibited a greater propensity for visual sampling of images via self-paced actions (i.e., keypresses) during systole^14^. Similar effects were observed in a study examining the relationship between the cardiac cycle and self-initiated movements in both action execution and action observation contexts^15^. In addition, Konttinen et al. reported that nonelite rifle shooters were more likely to pull the trigger during a cardiac window largely overlapping with the systolic phase of the cardiac cycle^16^. In a study examining cardiac modulation of the startle reflex, Schulz et al. found that the motor execution component of response time was accelerated during the early phase of the cardiac cycle^17^. Using a battery of cognitive control tests, Makowski et al. further demonstrated that systole was associated with faster simple reaction times and a higher rate of action inhibition failures compared with diastole^18^. Supporting impaired inhibitory motor control at systole, Ren and colleagues reported prolonged stop-signal reaction times when stop cues occurred during systole, accompanied by reduced amplitudes of the stop-signal P3 (i.e., an electrophysiological index of inhibitory processing)^19^. Notably, this effect was independent of the stop cue being simple and non-emotional, or complex and emotional. In a subsequent study, the same research group showed that when task-irrelevant information was coupled to cardiac phase, systole trials were associated with significantly shorter stop-signal reaction times and enhanced N2 amplitudes, indicating greater cognitive control^20^. In both studies^19,20^, increased amplitudes of the heart-evoked potential (i.e., a cortical marker of interoceptive attention to heartbeats) during systole were linked to enhanced suppression of systole-bound task-irrelevant information, thus improving behavioural performance. Interestingly, the notion of action facilitation during systole is conceptually supported by empirical observations indicating that risk-prone decision-making is increased during this phase of the cardiac cycle^67^.

Heightened activity of the motor system at systole has also been documented for oculomotor processes^12,13^. Ohl et al. reported that, during a visual fixation task, the generation of involuntary microsaccades was increased during systole, thereby extending evidence for motor facilitation to oculomotor behaviour^12^. Relatedly, Galvez-Pol et al. showed that, in a free visual search task, more saccadic eye movements were generated during the systolic phase^13^.

Evidence of motor invigoration at systole contrasts with findings indicating that that sensory perception including auditory^43,52^, and somatosensory^44–47^ stimuli, is attenuated during systole. It has been proposed that cardiac-related inhibition of behaviourally irrelevant sensory information may facilitate responding to environmental stimuli^14,68^. Although processing of motivationally salient stimuli is enhanced at systole^36–39^, we found no evidence that action invigoration was linked to appetitive or aversive motivational action biases. Notably, much of prior research work examining emotion perception and appraisal in relation to the cardiac cycle has relied on emotional probes that do not directly engage reward processing mechanisms.

In contrast to our findings, Rae et al. temporally aligned the delivery of stop signals in a conventional stop signal task with different phases of the cardiac cycle and demonstrated stronger response inhibition during systole than diastole, as indicated by shorter stop signal reaction times and longer stop signal delays at systole^21^. This effect was replicated in an independent study^22^. In a subsequent investigation, the same authors employed a modified version of a go/nogo task to probe reactive and intentional response inhibition. The task included a higher proportion of go trials to elicit a prepotent go response that participants were required to suppress on nogo trials (i.e., reactive inhibitory control)^24^. Moreover, they incorporated ‘choose’ trials where participants could freely decide whether to act or withhold a response (i.e., intentional inhibitory control). No significant effects of cardiac phase were observed for both reactive and intentional response inhibition^24^. Building on this work, Mussini et al. further adapted the modified go/nogo paradigm to enhance task engagement and uncertainty. They found that diastole was associated with a significantly greater proportion of cue-initiated volitional actions during ‘choose’ trials, an effect that was robust to physiological (i.e., increased heart rate) and psychological (i.e., heightened stress) manipulations^23^. Moreover, in a separate EEG study, the same authors demonstrated that greater expectancy of proactive inhibition during motor preparation was linked to slower reaction times, reduced readiness potential and enhanced heart-evoked potential^25^. In contrast, two studies employing the Libet’s clock task reported null findings, observing no statistically significant cardiac phase-dependent bias in voluntary action initiation^26,27^. However, using a modified version of the Libet’s task, Germanova et al. showed that participants’ detection of the ‘urge to move’ was predominantly aligned with the diastolic phase of the cardiac cycle^28^. Taken together, the observed divergent findings may be attributable to methodological inconsistencies across studies, including differences in experimental paradigms, cardiac signal acquisition methods, and the operational definitions of cardiac events.

The results of the exploratory fMRI analysis were intriguing and suggested that the behavioural and computational evidence for comparatively stronger action invigoration at systole is associated with reduced neural activity supporting action inhibition. Notably, this interpretation aligns with the behavioural observation that increased go responding at systole was most pronounced on nogo trials. However, the findings of the exploratory fMRI analysis are preliminary and should be treated with caution as they did not survive multiple comparison correction.

The observation of greater peak BOLD activity in the right IFG during diastole is consistent with the established functional neuroanatomy of inhibitory control^69–71^. Lesion and neuroimaging studies have identified the right IFG as a key hub implicated in cognitive processes such as response inhibition (which requires the suppression of an intended movement), and task-set switching (which requires the inhibition of competing stimulus-response mappings during task switching)^70^. Response inhibition is thought to be implemented via fronto-subthalamic circuitry, exerting downstream modulatory effects on the motor cortex^69^. Of note, the dorsomedial prefrontal cortex identified in the inaction contrast has also been implicated in inhibitory action control^72^.

The predictive coding framework provides a helpful theoretical framework to interpret cardiac effects on action and perception^19,20^. Within a predictive coding framework, these findings suggest that cardiac phase-dependent interoceptive signals modulate action selection by altering the precision of motor priors rather than the valuation of outcomes^73,74^. Phasic baroreceptor signalling at systole may transiently increase the precision weighting of prepotent action policies, biasing behaviour toward action execution independently of motivational valence. Consistent with this account, systole was associated with an affect-independent increase in go responding and elevated go-bias parameters, with no evidence for modulation of Pavlovian action biases. This interpretation accords with theoretical and empirical work showing that interoceptive signals shape behavioural control through precision modulation^75,76^.

This study has several important limitations. As a secondary analysis of an existing dataset, we took a naturalistic, *post hoc* approach to examining the effects of cardiac phase on action selection. While informative, this approach precludes definitive directional inferences regarding the relationship between cardiac activity and behaviour (i.e., whether changes in behaviour influence cardiac dynamics or vice versa). Prospective designs incorporating a priori time locking of task events to specific cardiac phases may help address this limitation, although such approaches can be challenging to implement due to unwieldly designs. As recently highlighted^40^, the field would benefit from a standardized consensus definition of systole and diastole to facilitate comparability of findings across studies. Following a physiologically defined approach allowed us to capture the full period of ventricular contraction; however, this broader window necessarily includes early post-R-peak intervals that may precede the peak cortical expression of arterial baroreceptor signalling typically reported in human EEG studies (150–250 ms post-R-peak). Accordingly, our results should be interpreted as indexing cardiac phase as a physiological state during ventricular contraction, rather than isolating the precise cortical arrival window of baroreceptor-specific signalling. Future research should continue to reconcile these physiological definitions with estimated-latency models to further refine our understanding of cardiac-brain temporal dynamics.

A further limitation of this study is the pronounced sex imbalance, which may limit generalisability. Sex differences in cardiovascular autonomic regulation and interoceptive processing are well documented and may influence behavioural and neural effects dependent on cardiac phase^77,78^. In addition, sex differences in inhibitory control could further modulate cardiac-related effects on action selection^79^. Future studies should therefore include more balanced samples and consider sex and hormonal status explicitly.

While the orthogonalized go/nogo task is a well-established and validated paradigm for assessing the effects of valence and action requirement on decision-making, we acknowledge that its sensitivity to detect subtle cardiac–valence interactions has not been empirically established.

In conclusion, this study extends existing evidence on the relationship between physiologically defined cardiac phase and action decisions. The findings of this study provide preliminary support for the notion that cardiac systole is linked to stronger go responding, independent of appetitive or aversive motivational action biases, and may be underpinned by diminished neural activity related to action inhibition. Future research should seek to replicate and extend the findings of this study using designs that incorporate a priori time locking of task events to the cardiac phase and non-invasive brain stimulation techniques to probe the casual role of cardiac-related neural mechanisms of action decisions.

## Supplementary Information

Supplementary Information.

## Data Availability

The data presented in this paper will be made available upon request to Filippo.Queirazza@glasgow.ac.uk.

## Code availability

The code used for statistical and computational modelling will be made available upon request to Filippo.Queirazza@glasgow.ac.uk .
