# Supplementary material for: Cardiac systole is associated with enhanced go responding in an orthogonalized go/nogo task
Source: Sci Rep. 2026 May 21;16:23408. doi: 10.1038/s41598-026-52930-9 (PMC13408127; doi:10.1038/s41598-026-52930-9)
Supplement: Supplementary file 1 — Supplementary Information. [file 41598_2026_52930_MOESM1_ESM.docx]

**
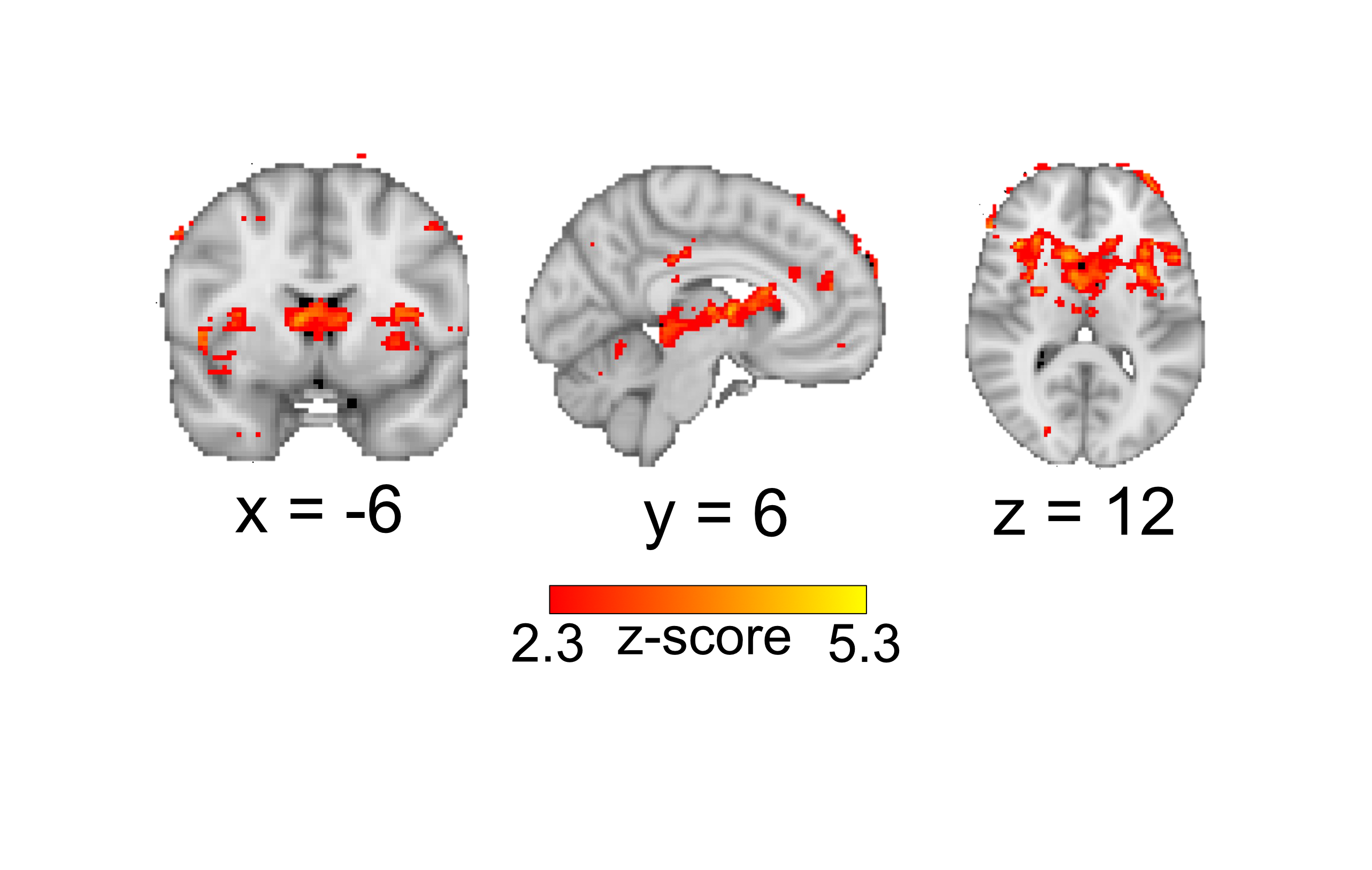
**

**Supplementary Figure 1. Cardiac contrast.** Brain activity reflecting underlying pulsatile cardiac activity in the cerebrospinal fluid, brain stem, in the vicinity of large blood vessels at the edge of the brain parenchyma and in interoceptive brain areas such as the anterior cingulate cortex and insula. MNI coordinates are shown.
